# Using electronic medical records to analyze outpatient visits of persons with epilepsy during the pandemic—experience from a low middle income country

**DOI:** 10.1186/s42494-024-00192-1

**Published:** 2025-01-15

**Authors:** Rajeswari Aghoram, Pradeep P. Nair, Anudeep Neelagandan

**Affiliations:** 1 Neurology, Super Specialty Block, Jawaharlal Institute of Postgraduate Medical Education and Research, Pondicherry, 605006 India; 2 Hospital information systems, Jawaharlal Institute of Postgraduate Medical Education and Research, Pondicherry, 605006 India

**Keywords:** Electronic medical records, Manual validation, Interrupted time series analysis

## Abstract

**Background:**

Electronic medical records (EMR) can be utilized to understand the impact of the disruption in care provision caused by the pandemic. We aimed to develop and validate an algorithm to identify persons with epilepsy (PWE) from our EMR and to use it to explore the effect of the pandemic on outpatient service utilization.

**Methods:**

EMRs from the neurology specialty, covering the period from January 2018 to December 2023, were used. An algorithm was developed using an iterative approach to identify PWE with a critical lower bound of 0.91 for negative predictive value. Manual internal validation was performed. Outpatient visit data were extracted and modeled as a time series using the autoregressive integrated moving average model. All statistical analyses were performed using STATA version 14.2 (Statacorp, USA).

**Results:**

Four iterations resulted in an algorithm, with a negative predictive value 0.98 (95% CI: 0.95–0.99), positive predictive value of 0.98 (95% CI: 0.85–0.99), and an F-score accuracy of 0.96, which identified 4474 PWE. The outpatient service utilization was abruptly reduced by the pandemic, with a change of -902.1 (95%CI: -936.55 to -867.70), and the recovery has also been slow, with a decrease of -5.51(95%CI: -7.00 to -4.02). Model predictions aligned closely with actual visits with median error of -3.5%.

**Conclusions:**

We developed an algorithm for identifying people with epilepsy with good accuracy. Similar methods can be adapted for use in other resource-limited settings and for other diseases. The COVID pandemic appears to have caused a lasting reduction of service utilization among PWE.

**Supplementary Information:**

The online version contains supplementary material available at 10.1186/s42494-024-00192-1.

## Background

The COVID-19 pandemic upended traditional care provision pathways worldwide, including India, and diverted important resources and focus from various non-communicable diseases such as epilepsy [1]. Several studies during the pandemic reported increased anxiety and stress levels among persons with epilepsy (PWE), as well as difficulties in accessing care and use of tele-health services. These studies were mostly conducted online and in high-income countries. [2, 3]

Careful measurement of the effects pandemic-induced restrictions is the key to understanding the full consequences of the pandemic and to better prepare health systems [4]. Quantifying the impact of pandemic-induced disruptions is challenging in low- and middle- income countries (LMIC) due to the lack of reliable and high-frequency data measuring various health outcomes. In this context, electronic medical records (EMR) can provide rich data that can be analyzed using both traditional and non-traditional methods to yield important insights [5]. However, the data quality of the EMR in LMIC is often suboptimal. Though established vocabularies are available and can be mapped to various coding systems like International Classification of Diseases (ICD), mapping them in the setting of the EMR in LMIC is often challenging. Other challenges in using EMR data include a lack of training and non-uniform data capture. [6]

In this study, we report the processes used, the challenges faced and the lessons learnt while developing and validating an algorithm for identifying PWE from our EMR. Using this algorithm, we extracted and analyzed the outpatient service utilization of PWE during the pandemic and after.

## Methods

### Setting and database

Our institute is a publicly funded institute of national importance providing tertiary care in the southern region of India. The institute uses proprietary electronic medical record software developed by *Softlink Inc* on ASP.Net MVC platform. This physician-hosted system has in-house servers that store the data. It is a relational database where each patient’s data is stored under a unique identifier. Our institute started using this system in 2015. The department of neurology has employed a unique modular interface within this EMR to document all outpatient interactions since 2017. In India, the first lockdown due to the pandemic was declared at the end of March 2020. By May 2020, we had implemented a telemedicine service to provide continued care for PWE. In January 2022, all COVID-associated curbs were lifted, telemedicine services were discontinued and all routine hospital services were resumed.

### Algorithm development

After obtaining ethics approval (JIP/IEC-OS/2021/303), we included all relevant data of neurology captured between January 2018 and December 2023. We segmented the data into three time periods: January 2018 to March 2020 (27 months) as the pre-pandemic period; April 2020 to December 2021(20 months) as the pandemic period, and January 2022 to December 2023 (24 months) as the post-pandemic period. A preliminary search of the database using standard disease codes for epilepsy and seizures did not yield any results. So, we used an iterative development cycle consisting of design, writing, implementation, and testing phases to develop the algorithm. In each cycle, we used and updated a logic grid (Supplementary Table S1) to identify key components of the case definition. A three-member team, consisting of a domain expert (epileptologist), a clinician and an information specialist, populated the logic grid and developed the algorithms using structured query language (SQL). At the end of each cycle, the algorithm was run on the database, and results were extracted for validation. There was no pre-processing of data involved. Duplicate records were removed using Excel and confirmed manually. Missing data was considered equal to null value. The results of the validation were reviewed by the three-member team to update the logic grid and tweak the algorithm.

### Validation

We used the methods proposed by Liu et al. to estimate sample sizes for validation studies of electronic phenotyping [7]. As the data pertains to tertiary care referral neurology services, we assumed the prevalence of epilepsy in this group to be 30% [8], which is considered a moderately common disorder. To achieve a critical lower bound of 0.91 for the negative predictive value (NPV), we aimed to develop a sensitive algorithm to serve our intent to explore the health-seeking practices of PWE. For validation, we needed 100 individuals with epilepsy and 100 individuals without epilepsy [7]. One treating clinician with more than five years’ experience performed the validation by direct chart review of electronic medical records along with neuroimaging and electroencephalography (EEG). This validation set was compared with the results of each iteration of the algorithm to calculate various performance metrics. All algorithm search results were converted into a CSV file and exported into Excel (Richmond, USA). We estimated sensitivity, specificity, positive predictive value (PPV), and NPV using standard formulae and calculated their 95% confidence intervals (CI) using the exact binomial method. In addition, we calculated the F-score, which is a single measure of test performance and is defined as the harmonic mean of PPV and sensitivity [9]. F-scores range from 0 to 1, with higher scores indicating better performance. We identified the algorithm with highest F-score and NPV as our final algorithm.

### Data extraction

The algorithm with maximum NPV and highest F-score was used to extract monthly outpatient visit data of PWE over a period from January 2018 to December 2023. The data of PWE with two or more visits during the pre-pandemic period was extracted untill March 2023 and used for the model development. We defined a telemedicine user as a PWE who had any visit (doctor consult or prescription refills) during the pandemic period.

### Model development

We used the autoregressive integrated moving average (ARIMA) model for performing interrupted time series analysis of the outpatient visit data. Stationarity was checked for using augmented Dickey-Fuller unit root test and the first differential was found to be acceptable. The autoregressive and moving average parameters were estimated using the autocorrelation and partial autocorrelation plots of pre-pandemic period visit data. Different models were compared in terms of the values of coefficients and their significance, volatility determined by sigma square, log likelihood, and information criteria (Akaike and Bayesian). Model residuals were checked for autocorrelation using graphs and the portmanteau test for white noise. This model was then used to forecast visit data from April 2023 to December 2023 and compared with actual data. The time series graph suggested both a level change and slope change of the visit data due to the pandemic, so we used both step and ramp transfer functions. Dummy variables for step change, with values of 1 after the pandemic (April 2020) and 0 before the pandemic, as well as for ramp change represented by the months since the onset of the pandemic were used as transfer functions to model the effect of pandemic. All statistical tests were carried out using STATA version 14.2 (STATAcorp LP, College Station, TX, USA).

## Results

**Fig. 1:**
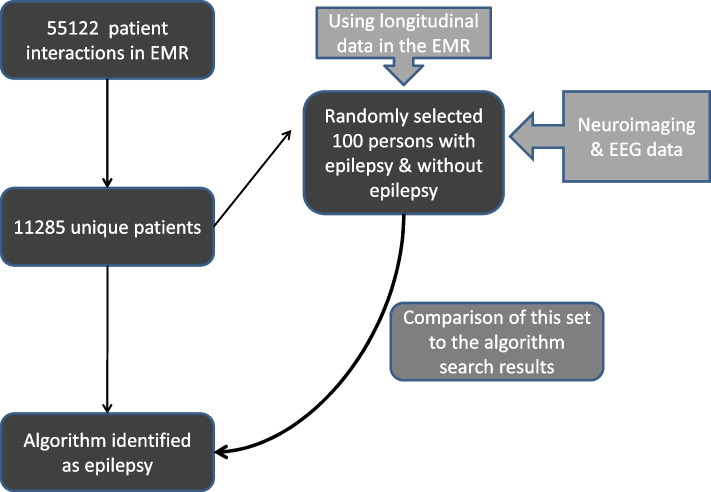
Flow of patient records during algorithm development

The Fig. 1 depicts the flow of patient records during the process and the final algorithm is presented in the supplemental material (Supplementary Table S2).

We found the last iteration (Table 1) had a NPV with a lower bound of 95% CI above 0.91 and exhibited the best accuracy (F-score = 0.96). We stopped further development at this point and accepted it as the final algorithm. Using this algorithm, we found the prevalence of epilepsy in our database to be 38.9% (95%CI 38.1–39.8%).

**Table 1:** Results of the validation of algorithms

Algorithm used	Algorithm identified	Validated diagnosis of epilepsy (*n* = 100)	Validated diagnosis of no epilepsy (*n* = 100)	Positive predictive value (95%CI)	Sensitivity (95% CI)	Negative predictive value (95% CI)	Specificity (95%CI)	F Score
Drugs (iteration 1)	Yes	24	0	100%	24% (16–33)	75% (73–77)	100% (96–100)	0.39
	No	76	100					
Keywords (iteration 2)	Yes	86	6	86% (74–93)	86% (78–92)	94% (91–96)	94% (87–98)	0.86
	No	14	94					
Both (iteration 3)	Yes	87	7	84% (72–91)	87% (79–93)	94% (91–97)	93% (86–97)	0.856
	No	13	93					
Final algorithm (iteration 4)	Yes	95	1	98% (85–100)	95% (89–98)	98% (95–99)	99% (95–100)	0.96
	No	5	99					

All confidence intervals estimated using binomial exact method; F scores = $\frac{2(\text{Positive predictive value}+\text{sensitivity})}{\text{Positve predictive value}*\text{sensitivity}}$

Of the 4474 PWE, 3008 (1342 women, 44.6%) had two or more visits pre-pandemic. Among them, 1588 (52.8%; 95%CI: 51–54.6%) used telemedicine services. We found women were less likely to use telemedicine services (OR: 0.83; 95%CI: 0.72–0.96; Supplementary Table S3); but no differences across various age groups were observed.

**Fig. 2:**
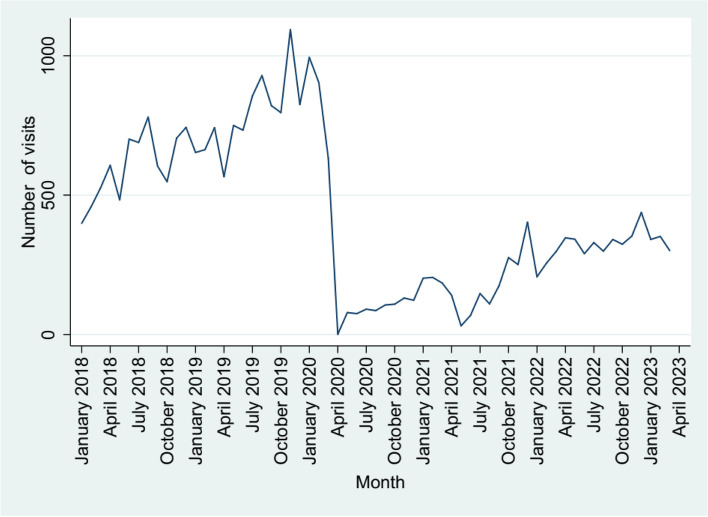
Time series graph of monthly out patient visits between January 2018 and March 2023

**Fig. 3:**
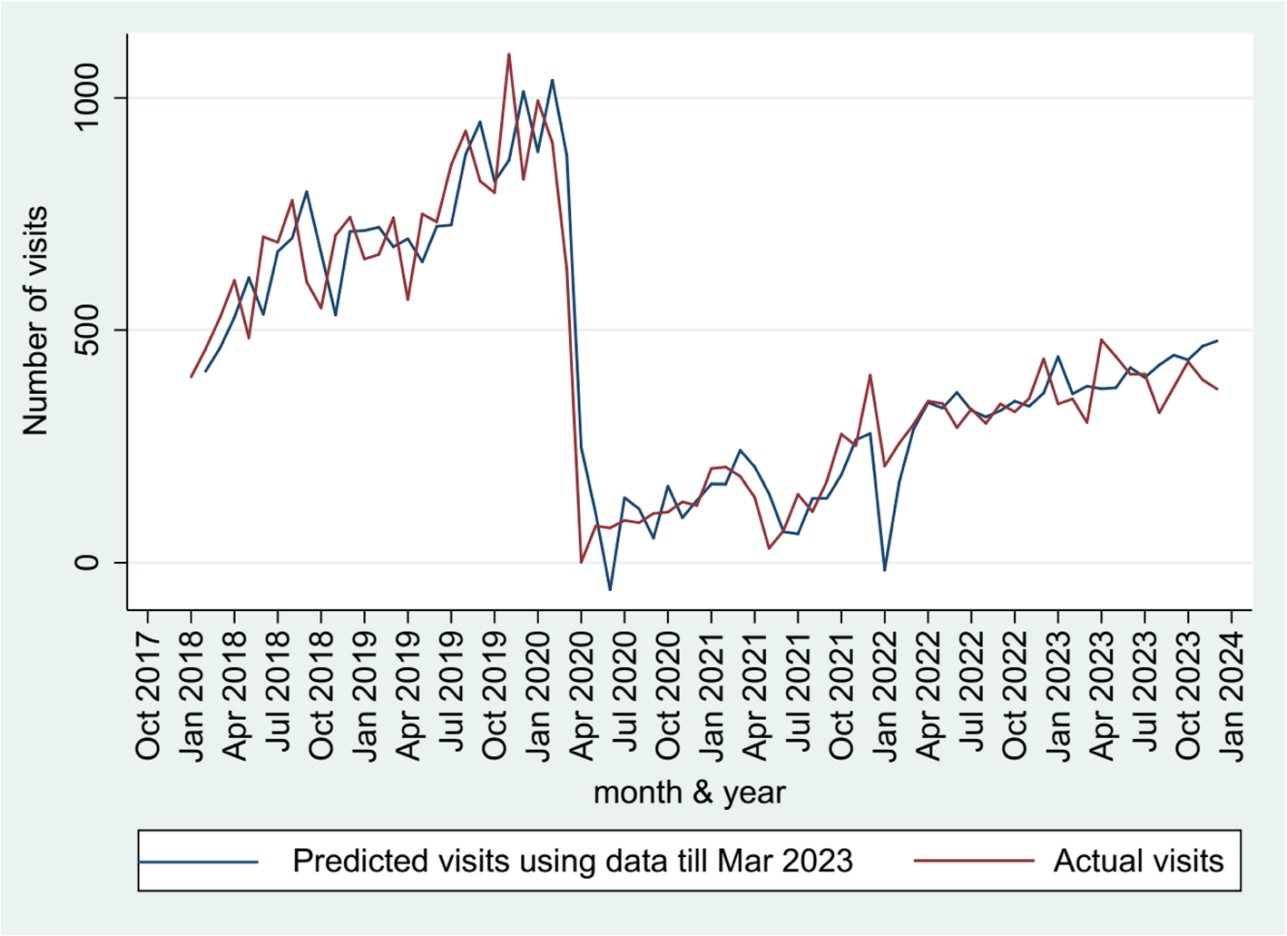
Time series graph of out patient visits predicted from the ARIMA model and actual between January 2018 and December 2023

A time-series graph of outpatient department (OPD) service utilization until March 2023 is shown in Fig. 2. The ARIMA model with four autoregressive terms, two moving average terms of the first differential without seasonal changes was identified as most optimal. We then added the transfer functions and modeled the data up to March 2023. We found that the pandemic resulted in both a sudden decline in the number of visits (step transfer function: -902.1; 95%CI: -936.55 to -867.70) and slow recovery (ramp transfer function: -5.51; 95%CI: -7.00 to -4.02) (Supplementary Table S4 and Figure S1). We used this model to predict visit data from April to December 2023. The predictions of this model were closely aligned to the actual data (Fig. 3), with a median error of -3.5%.

## Discussion

We described the methods to develop an algorithm to identify PWE from the EMR, with an F-score of 0.96, indicating high accuracy. During the pandemic, telemedicine consultations were utilized by 52.8% (95%CI: 51–54.6%) of PWE. The time series model suggests that the pandemic had an immediate and sustained effect on reducing OPD care utilization by PWE.

### Algorithm development and validation

The nature of the EMR data and how it is captured has a bearing on the nature of algorithm necessary to extract data. Using a database generated by pooling data from different general practice databases in Canada, and an approach similar to ours, Williamson and colleagues reported a higher NPV 99.9% (99.7–100%) but a lower PPV of 85.6% (95%CI:80.2–91.1) [10]. A systematic review of studies on various algorithms for epilepsy diagnosis, using a combination of symptom and diagnosis codes with or without anti-seizure medications on administrative data, reported overall high sensitivity (88.6%; 95%CI: 85.7– 91.0%) and specificity (97.9%; 95% CI: 98.5–96.9%) [11]. In our study, we used simple free text searches instead of codes to achieve similar results. Evidence suggests that using free text searches may improve algorithm performance, and the relationship between text and codes as entered by physicians, as well as the impact of codes selection on search algorithms, are not well understood [9, 12]. The Electronic Medical Records and Genomics (eMERGE) study has also employed a similar approach [13]. A systematic review of studies on algorithms, ranging from simple text searches to natural language processing algorithms to detect named clinical condition, showed that addition of free text searches improved sensitivity (median sensitivity of 78% (codes + text) vs 62% (only codes); *P* = 0.03) [9]. Specifically for epilepsy, a regularized logistic regression model with extreme gradient boosting was trained to identify PWE from pooled outpatient data of nine hospitals. It was reported that using 285 features, including 246 keywords, the model demonstrated good performance (area under the receiver operating characteristic curve of 1; 95% CI = 0.99–1.00) [14]. In a resource-constrained setting, we utilized a simple text mining approach with an expert team to enhance algorithms, which allowed us to reduce computational requirements while maintaining similar performance.

The LV Prasad Eye Institute network in India has developed an in-house EMR system called “eyeSMART” [15] and has used it to analyze various eye conditions [16]. They utilize an app-based standard format for recording all data and have mapped ICD codes for the diagnosis, but they have not validated any diagnoses [16]. In contrast, our EMR application does not have a standard format for data capture (except for medication prescription) or automated code mapping and is designed to accommodate all broad specialties. Additionally, we believe that the variability of data in neurology is greater than in ophthalmology, which makes it challenging to establish a standard format for entries and code mapping. Thus, we are unable to comment on the relative merits of their approach compared to ours.

Validation is necessary to understand how accurately an algorithm captures the target condition, as it strongly affects the reliability of subsequent observational studies performed using this algorithm [17]. Since there is no single gold standard, multiple methods of validation have been described. [17, 18] We performed a manual validation, which is a method similar to that used for the identifying patients with multiple sclerosis [19]. This approach also allowed us to calculate all relevant metrics [9] and choose the best algorithm for our purposes. Another way to validate the diagnosis is by estimating the proportion of those identified by the algorithm and comparing it to available data. Our algorithm estimated the proportion of PWE in our neurology specialty EMR to be 38.9% (95%CI 38.1–39.8%). This is comparable to the proportion reported by other neurology specialty clinics providing tertiary care. [8] We did not perform external validation due to logistic and data-sharing issues. To the best of our knowledge, we report here the first validation study for identification of PWE from EMR data in a low- and middle-income country.

### Influence of the COVID pandemic

A systematic review identified 81 studies on healthcare utilization during the pandemic, with only four from LMIC. It reported that an overall median reduction by 1/3 for all services and 42% (95%CI: 32–53%) for outpatient services [20]. A study using health databases from multiple regions in Ethiopia demonstrated that the pandemic led to a decrease in hospital services utilization that mirrored the intensity of infection, noting a differential effect based on the nature of services [21]. In India, the second wave of the pandemic, which occurred between March 2021 and June 2021, was more intense; however, we did not observe as significant an impact on visits as during the initial lockdown. Furthermore, we show that despite the easing of restrictions, outpatient service utilization is only showing a slow recovery.

It has been suggested that the reduction in service utilization during the pandemic may be merely due to a decrease in unnecessary service utilizations [20]. On the other hand, India already has a high treatment gap for epilepsy [22]. There is evidence suggesting that access to care for PWE was compromised by the pandemic across the world. A systematic review of anti-seizure medication use among PWE during the pandemic reported that there were changes in compliance due to a lack of follow-up and difficulties in accessing medications [23]. Another study utilizing drug dispensing databases in Germany noted a decrease in the number of newly diagnosed epilepsy cases during the pandemic, suggesting that individuals were unable to access services [24]. A study using emergency department data for seizure visits in the United States concluded that there was a reduction in visits from March to December in 2020,but this returned to pre-pandemic levels by mid-2021 [25]. Similarly, in Italy, a report indicated that the use of anti-seizure medication and access to emergency department for seizures fell during the lockdown. However, there was an increase in both metrics post-pandemic, as shown through interrupted time series analysis.[26]. Our results suggest not only a decline but also a poor recovery of service utilization among PWE. Poor recovery of OPD utilizations has also been documented in other settings. [27, 28] A systematic review of the effects of COVID pandemic on Indian healthcare utilization identified seven studies in areas such as eye care, nephrology, trauma care and cancer care,[29] but no studies on epilepsy. All identified studies showed a significant reduction in the in utilization of services during the pandemic [29]. Although these studies did not evaluate any single service but rather a package of services, did not use robust methodologies like interrupted time series analysis, and did not evaluate a post-pandemic period [29],they all suggested reduced service utilization during the pandemic. Access to healthcare has been reported as one of the most important determinants of healthcare utilization for PWE in previous studies [30]. Furthermore, a widening treatment gap for mental health disorders like depression and anxiety has been reported [31]. A study on epilepsy care in resource-limited settings during the pandemic, based on a survey of healthcare professionals, concluded that COVID-induced challenges resulted in significant disruption of epilepsy care, particularly in LMIC [32]. Thus, we are inclined to believe that this reduction in service utilization may be more indicative of the widening treatment gap following the pandemic rather than a reduction in unnecessary utilization. However, larger community-based studies are needed to confirm this.

Our study has certain strengths and limitations. We have described methods to leverage EMR in resource-limited settings, even with poor coding practices. Though our EMR software is proprietary and our algorithm may not be directly applicable to other settings, we believe the methods described can be applied to any setting and/or disease. Furthermore, validation studies from the eMERGE network [13] and the Canadian Primary Care Sentinel Surveillance Network (CPCSSN) [10] have shown the interoperability of algorithms with minor tailoring across different EMR systems. We used data only from outpatient services at a single hospital and may have missed those patients who have migrated to other service providers. However, it remains pertinent that the service utilization has reduced and has yet to fully recover. Additionally, we do not have outcome data for these patients, thus we cannot fully access how this reduced service utilization has affected the PWE.

## Conclusions

A combination of anti-seizure medications and free text searches provided optimum performance in detecting PWE in a neurology specialty database. The pandemic-induced disruptions seem to have a persistent effect on outpatient care utilization by PWE, resulting in a slow recovery. The methods described in this paper can be employed to generate high-quality data to inform health system strengthening.

## Supplementary Information

Supplementary Material 1.

Supplementary Material 2.

## Data Availability

Data has been deposited in the ICMR repository and can be made available on reasonable request.

## Abbreviations

EMR: Electronic medical record
ICD: International classification of disease
LMIC: Low-middle income countries
NPV: Negative predictive value
PWE: Persons with epilepsy
PPV: Positive predictive value
