# Supplementary material for: Using electronic medical records to analyze outpatient visits of persons with epilepsy during the pandemic—experience from a low middle income country
Source: Acta Epileptol. 2025 Jan 15;7:6. doi: 10.1186/s42494-024-00192-1 (PMC11960259; doi:10.1186/s42494-024-00192-1)

**Supplement 1**

TableS1: Logic grid for case definition of epilepsy for algorithm development

|  | Keywords | Drugs | Exclusion | Number identified |
| --- | --- | --- | --- | --- |
| Iteration 1 | -- | Phenytoin, Valproate, phenobarbitone, levatericetam | Exclude trigeminal neuralgia | 376 |
| Iteration 2 | Seizure, epilepsy, post stroke seizures, Mesial temporal lobe sclerosis | -- |  | 3818 |
| Iteration 3 | Seizure, epilepsy, post stroke seizures, Mesial temporal lobe sclerosis | Phenytoin, Valproate, phenobarbitone, levatericetam | Exclude trigeminal neuralgia | 4139 |
| Iteration 4 | Seizure, Epilepsy | Phenytoin, Valproate, phenobarbitone, levetericetam | Non epilepsy clinic days | 4474 |

Table 1.2: Final SQL algorithm for case identification

| select distinct npw.CaseId, npw.IPNO, convert(varchar,cast(npw.entrydate as date),103) as VisitDate,  case when hp.PatientName is null then pfd.PatientName else hp.PatientName end as PatientName,  case when hp.Age is null then convert(varchar,pfd.Age)+' Years' else convert(varchar,hp.Age)+' '+hp.AgeIn end as Age,  case when hp.Sex is null then pfd.Sex else hp.Sex end as Gender,  from NeuroPatientWorklist npw  left join NeuroSummaryDiagnosis nsd on nsd.caseid=npw.CaseId  left join NeurologyHistory nh on nh.CaseId=npw.CaseId  left join HISPrescriptionMaster hpm on hpm.CaseID=npw.CaseId  right join HISPrescriptionDetails hpd on hpd.VoucherDate=hpm.VoucherDate and hpd.VoucherNo=hpm.VoucherNo  left join hispatient hp on hp.IPNO=npw.IPNO  where  npw.EntryDate<20200301 and datename(DW,convert(date,npw.EntryDate,101))='Friday'  **and**  ((nsd.DiagnosisDifferentials like '%seizure%' *or* nsd.DiagnosisDifferentials like '%seizure%disorder%' *or* nsd.DiagnosisDifferentials like '%epilepsy%') *or* (nsd.FinalDiagnosis like '%seizure%' *or* nsd.FinalDiagnosis like '%seizure%disorder%' *or* nsd.FinalDiagnosis like '%epilepsy%') *or* (nsd.Summary like '%seizure%' *or* nsd.Summary like '%seizure%disorder%' *or* nsd.Summary like '%epilepsy%') *or* (nh.ComplaintsAndDuration like '%seizure%' *or* nh.ComplaintsAndDuration like '%seizure%disorder%' *or* nh.ComplaintsAndDuration like '%epilepsy%')  **or**  (hpd.Description like '%valporate%' *or* hpd.Description like '%phenyton%' *or* hpd.Description like '%carbamezepine%' *or* hpd.Description like '%phenobarbitone%' *or* hpd.Description like '%levatericetam%'))  order by npw.IPNO, VisitDate |
| --- |

Table 1.3: Pandemic telemedicine visits of PWE by age and gender

| Gender | Children  (Age ≤18 years) N=699 | | Young adults  (19-40 years)  N=1699 | | Older adults  (41-59 years) N=486 | | Elderly  (≥60 years)  N=124 | |
| --- | --- | --- | --- | --- | --- | --- | --- | --- |
|  | Telemed users | Non-telemedusers | Telemed users | Non-telemedusers | Telemed users | Non-telemedusers | Telemed users | Non-telemedusers |
| Male | 244 | 182 | 468 | 381 | 159 | 144 | 43 | 45 |
| Female | 136 | 137 | 418 | 432 | 106 | 77 | 14 | 22 |
| Odds ratio (95%CI) | 0.74(0.54-1.01) | | 0.79(0.65-0.96) | | 1.24(0.85-1.83) | | 0.67(0.28-1.57) | |

Table S4: Results of ARIMA modelling


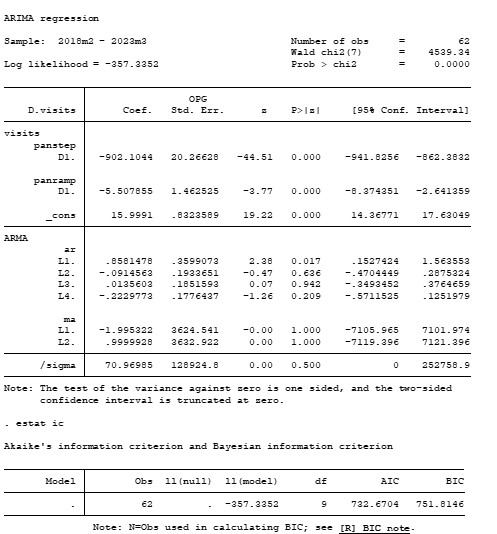


Figure S1: Model residuals


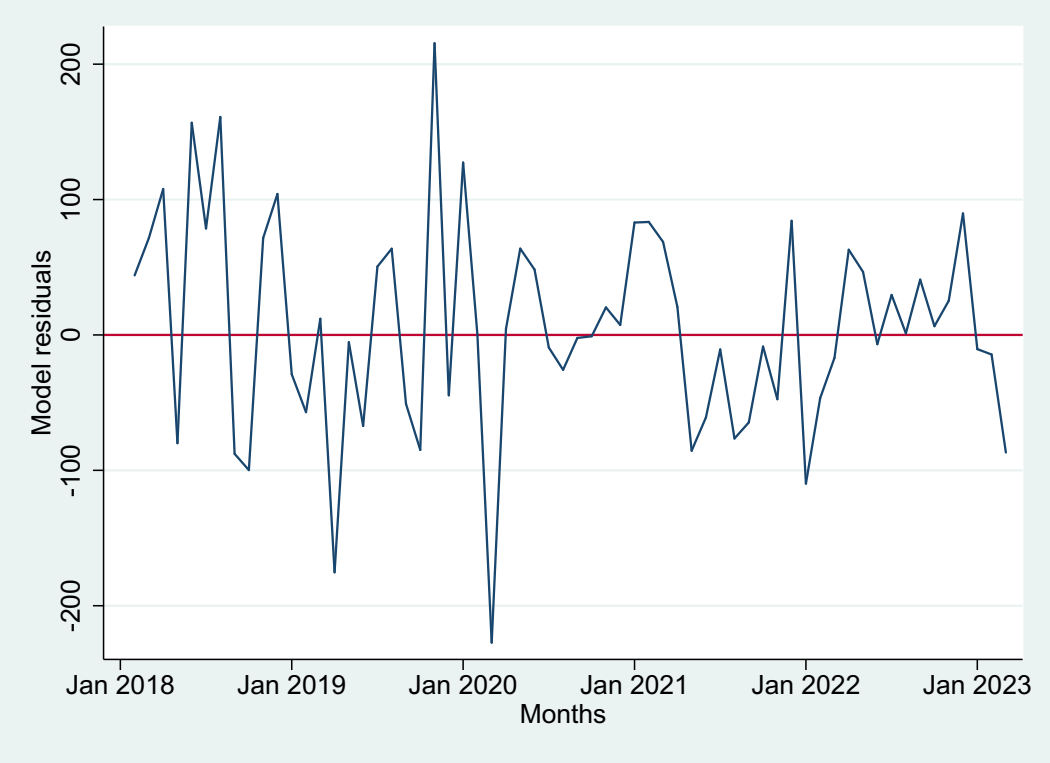

Supplement: Supplementary file 1 — Supplementary Material 1. [file 42494_2024_192_MOESM1_ESM.docx]
